# Molecular Dynamics Reveals Structural Consequences of Schizophrenia-Associated SLC6A1 Variants

**DOI:** 10.3390/ijms27219793

**Published:** 2026-10-31

**Authors:** Dmitrii A. Abashkin, Amal Abdurazakov, Ekaterina V. Marilovtseva, Ekaterina V. Semina, Yulia A. Chaika, Vera E. Golimbet

**Affiliations:** Russian Mental Health Research Center, Kashirskoe Sh., 34, Moscow 115522, Russia; abdurazakov.sci@yandex.ru (A.A.);

**Keywords:** schizophrenia, SLC6A1, GAT-1, GABA transporter, molecular dynamics, missense variants, deep learning, SCHEMA, AlphaMissense

## Abstract

The *SLC6A1* gene encodes the GAT-1 transporter, which mediates the reuptake of GABA in the central nervous system (CNS). Missense variants in this gene are associated with the development of neuropsychiatric disorders, including schizophrenia. Although in vitro assays demonstrate a loss of transport activity, they do not elucidate how specific amino acid substitutions disrupt local interactions within the protein. To delineate the structural consequences of these mutations, we analyzed the inward-open apo-state of human GAT-1 (PDB: 7Y7V) using microsecond-scale molecular dynamics (MD) simulations. This study compares the wild-type transporter with mutant forms, including the schizophrenia-associated variants R211C and A305T, alongside the A346S variant selected via deep learning-based pathogenicity predictors. The results demonstrate that these mutations do not globally destabilize the protein structure; rather, they induce specific rearrangements within local contact networks. The R211C substitution abolishes a hydrogen bond at the interface of extracellular loop 2 (EL2) and transmembrane helix 4 (TM4). A305T alters the TM1–TM6 interface adjacent to the sodium ion-binding site, and A346S strengthens TM1/TM6–EL4 interactions. The identified structural shifts suggest how pathogenic mutations remodel the GAT-1 interaction network and alter its conformational dynamics. These findings provide a foundation for subsequent molecular docking aimed at drug repurposing and for comprehensive MD modeling of the complete GAT-1 transport cycle.

## 1. Introduction

Schizophrenia is a biologically heterogeneous disorder involving complex neurodevelopmental abnormalities and aberrant neural network connectivity [1,2]. The GABAergic hypothesis attributes this cortical dysfunction to deficient inhibitory control between cortical interneurons and pyramidal cells. This deficit disrupts excitation–inhibition balance and cortical synchrony, altering information processing and downstream dopaminergic signaling [3,4]. Maintaining precise cortical inhibition requires the rapid clearance of γ-aminobutyric acid (GABA) from synaptic and extrasynaptic spaces by high-affinity transporters, primarily GAT-1 and GAT-3. The bulk of this clearance is mediated by GAT-1, a transporter encoded by the *SLC6A1* gene [5,6].

Missense variants in *SLC6A1* have been implicated in epilepsy, intellectual disability, and autism-spectrum disorder, frequently presenting as epilepsy with myoclonic-atonic seizures [7,8,9]. Rare *SLC6A1* variants have also been reported in schizophrenia sequencing cohorts, including damaging *de novo* missense variants and variants represented in the SCHEMA dataset [10,11,12,13,14,15]. Functional studies often distinguish defects in trafficking or surface expression from defects in the transport cycle, such as altered substrate binding, ion coupling, or conformational exchange [16,17,18,19,20]. However, these broad functional classifications fail to resolve the local interactions perturbed by a given substitution, nor do they establish when an occupancy change within limited MD sampling is large enough to support mechanistic interpretation.

GAT-1 is a Na^+^- and Cl^−^-coupled neurotransmitter:sodium symporter comprising 12 transmembrane helices. The first ten form the canonical LeuT fold, organized as two inverted pseudo-symmetrical five-helix repeats (TM1–TM5 and TM6–TM10), while TM11 and TM12 remain peripheral to the transport core. TM1, TM3, TM6, and TM8 form the central substrate-binding pocket, where the unwound segments of TM1 and TM6 directly coordinate GABA and the co-transported ions. GABA uptake operates via an alternating-access cycle in which the mobile bundle domain (TM1, TM2, TM6, and TM7) shifts relative to the scaffold domain (TM3–TM5 and TM8–TM10). Controlled by extracellular and intracellular gates, these structural rearrangements sequentially expose the binding site to the extracellular and cytosolic sides of the membrane [21,22,23].

Recent cryo-EM data have resolved multiple inward-facing states of this cycle. Human GAT-1 was captured in tiagabine-bound inward-open, apo inward-open, and GABA-bound inward-occluded conformations, alongside a GABA-bound cytosol-facing intermediate of rat GAT-1 [19,24,25]. Because outward-open conformations of GAT-1 have not been captured, reconstructing the complete conformational cycle still relies on structural templates from homologous LeuT-fold transporters.

This study evaluates three *SLC6A1* missense variants (Table 1). The p.Arg211Cys (R211C) variant was identified *de novo* in a schizophrenia patient via trio analysis by Rees et al. [10]. Functional screening demonstrated that R211C reduces GABA uptake, indicating a partial loss of GAT-1 function [18].

The p.Ala305Thr (A305T) variant was selected from the dataset of rare missense variants aggregated by the SCHEMA consortium [11]. A305T introduces a threonine hydroxyl group into the discontinuous segment of TM6, adjacent to the substrate-binding helices [19]. Functional assays of *SLC6A1* showed a pronounced decrease in GAT-1 activity and GABA transport for A305T; the variant was classified as a severe loss-of-function mutation based on in vitro data from HEK293T cells expressing the variant GAT-1 [18].

AlphaMissense assigned R211C and A305T pathogenicity scores of 0.723 and 0.976, respectively, reflecting distinct pathogenic potentials. To extend this comparison across a broader prediction range, we selected p.Ala346Ser (A346S, AlphaMissense score 0.113) as a likely benign reference variant [26]. This allowed us to assess the structural and dynamic consequences of substitutions spanning a gradient of predicted pathogenicity (Table 1). Although both substitutions introduce a hydroxyl group, A346S provides a structurally informative contrast to A305T due to their distinct locations, with A305T situated in the discontinuous segment of TM6 and A346S in EL4 between TM7 and TM8.

Although the functional consequences of several *SLC6A1* variants have been characterized using in vitro approaches, these assays primarily describe the final functional phenotype and do not directly elucidate the molecular events linking amino acid substitutions to impaired transport function [18]. For a secondary-active transporter such as GAT-1, functional activity is determined not only by protein stability or its plasma membrane expression but also by the coordinated dynamics of transmembrane helices, the opening and closing of intracellular and extracellular gates, the state of Na^+^- and Cl^−^-binding sites, the hydration of internal cavities, and the rearrangement of interaction networks facilitating GABA binding and translocation. Molecular dynamics (MD) simulations enable the investigation of these processes at atomistic resolution within an explicit lipid environment, making them a widely adopted approach that complements experimental methods in studying the structural-functional dynamics of membrane proteins [27,28]. Modern GPU-enabled GROMACS workflows make multi-replicate simulations of transporter-sized systems practical on workstation hardware [29,30,31,32]. In the context of genetic variants, one advantage of MD is the capacity to directly compare the conformational ensembles of wild-type and mutant proteins. This allows for the identification of local interaction changes that can propagate to functionally critical protein regions, even in the absence of pronounced global structural destabilization. This method has previously been applied to investigate neuropsychiatric disorder-associated missense mutations in GAT-1, elucidating how mutations within the transporter structure affect overall protein stability and ligand-binding capacity [33,34].

**Table 1:** External evidence and structural context for the three simulated substitutions.

Variant	Structural Location	External Evidence (Selection Rationale)	Reported Functional Evidence	AlphaMissense
R211C	EL2–TM4 boundary	*De novo * schizophrenia- associated variant [10]	Intermediate loss-of-function in a multi-variant uptake panel [18]; mild-reduction VUS (−36.8% vs. WT), below that study’s pathogenic threshold, in an iGABA fluorescence assay [35]	0.723 (likely pathogenic)
A305T	TM6a–TM6b discontinuity, adjacent to the primary substrate-binding site	Rare case-enriched SCHEMA missense variant [11]	Severe loss-of-function in the [18] functional series	0.976 (likely pathogenic)
A346S	EL4, between TM7 and TM8	Unreported variant; included for structural contrast with A305T	Was not functionally characterized	0.113 (likely benign)

In the present study, MD simulations and subsequent post-simulation analyses of conformational dynamics and hydrogen-bonding networks were employed to investigate the structural and dynamic consequences of selected missense mutations in the *SLC6A1* gene. This approach supplements existing experimental data with a mechanistic interpretation, enabling the identification of specific molecular alterations that potentially link individual amino acid substitutions to GAT-1 dysfunction.

## 2. Results

### 2.1. Variant Sites on the GAT-1 Map

The three sites sit in different parts of the fold (Figure 1). R211 is in EL2, immediately before TM4. A305 is in the unwound TM6 segment at the TM6a–TM6b break, next to the substrate site. A346 is in EL4 between TM7 and TM8.

Selection of the three alleles is summarized in Table 1. R211C removes a guanidinium at the EL2–TM4 boundary; A305T and A346S each add a hydroxyl in TM6 and EL4, respectively (AlphaMissense for A346S = 0.113, likely benign). The design compares packing and polar-contact responses across those environments; it does not treat the three substitutions as interchangeable disease labels.

### 2.2. Selection of Pore-Lining Residues

HOLE2 was used to map the permeation pathway as a series of maximally inscribed spheres along the pore axis. A residue was classified as pore-lining if at least one of its heavy atoms lay within 3.5 Å of a sphere surface, with the effective distance defined as the atom-to-sphere-center distance minus the local HOLE2 radius. That criterion recovers both transmembrane and loop contacts. For packing, we restricted the list to 30 constriction residues around TM1, TM3, TM6 and IL4 (58–61, 63–66, 69–70, 136, 140, 294–297, 300–303, 305–306, 309, 396–397, 399–400, 402–403 and 406). All C(30,2) = 435 unordered pairs were screened; 133 pairs with a minimum heavy-atom distance < 4.5 Å on the 200–1000 ns trajectories entered the packing panel. Those pairs cluster at the TM1–TM6 interface, including position 305. The fixed constriction region of interest (ROI) contains no pairs at or near R211 or A346, so the packing layer cannot report contact change at those two sites.

### 2.3. Soft Effect-Size Lists Against the Permutation-Derived Chance Floor

Cohen’s *d* was calibrated against its null at the replicate size used everywhere (*n*_1_ = *n*_2_ = 4) before any contact-drift interpretation. For each residue pair, *d* was computed from replicate-level occupancies as

(1) $$d=\frac{{\bar{O}}_{\mathrm{mut}}-{\bar{O}}_{\mathrm{WT}}}{s_{p}},s_{p}=\sqrt{\frac{(n_{\mathrm{mut}}-1)s_{\mathrm{mut}}^{2}+(n_{\mathrm{WT}}-1)s_{\mathrm{WT}}^{2}}{n_{\mathrm{mut}}+n_{\mathrm{WT}}-2}}.$$

Here, *d* is Cohen’s standardized mean difference, and *O* denotes replicate-level contact occupancy; the overbar denotes the mean across replicates; the subscripts mut and WT identify the mutant and wild-type systems, respectively; *s_mut_* and *s_WT_* are the corresponding sample standard deviations; *n_mut_* and *n_WT_* are the replicate counts; and *s_p_* is the pooled standard deviation.

For the pooled-variance two-sample comparison, the same standardized difference relates to Student’s t by

(2) $$t=\frac{{\bar{O}}_{\mathrm{mut}}-{\bar{O}}_{\mathrm{WT}}}{s_{p}\sqrt{\left(\frac{1}{n_{\mathrm{mut}}}+\frac{1}{n_{\mathrm{WT}}}\right)}}=d\sqrt{\frac{n_{\mathrm{mut}}n_{\mathrm{WT}}}{n_{\mathrm{mut}}+n_{\mathrm{WT}}}}.$$

$\sqrt{2}\sqrt{2}$ Here, *t* is the equal-variance two-sample Student’s *t* statistic. With *n_mut_* = *n_WT_* = 4, *t* = *d* and *d* = *t*/, giving 6 degrees of freedom. In Equations (3) and (4), *P* denotes a two-sided null-tail probability and *t*_6_ denotes the corresponding Student’s *t* variate. An absolute *d* cutoff therefore maps onto a two-sided t tail. For the conventional soft threshold,

(3) $$P\left(\left|d\right|\geq 0.8\right)=P\left(\left|t_{6}\right|\geq 0.8\sqrt{2}\right)\approx 0.301.$$

About 30% of null comparisons then exceed |*d*| = 0.8 (Figure 2a). Among the 133 packing pairs retained for heavy-atom occupancy (≤4.5 Å), this null rate corresponds to 133 × 0.301 ≈ 40 soft selections per contrast. At the hard threshold,

(4) $$P\left(\left|d\right|\geq 3\right)=P\left(\left|t_{6}\right|\geq 3\sqrt{2}\right)\approx 0.0054,$$

corresponding to approximately 133 × 0.0054 ≈ 0.7 expected pairs.

To evaluate the six pairwise packing contrasts across the 133 structural pairs, we initially applied a soft threshold of |*d*| ≥ 0.8. Under the analytic 4-versus-4 null model (Figure 2a,b), 30.1% of pairs (approximately 40 of 133) are expected to exceed this threshold by chance. The observed counts for the three variant-versus-WT contrasts (46 for WT–A305T, 34 for WT–R211C, and 34 for WT–A346S) track close to this random expectation rather than clearly exceeding it. Cross-variant comparisons yielded comparable soft counts (33 for R211C–A305T, 51 for R211C–A346S, and 63 for A305T–A346S).

Because the numbers of packing contacts selected at the soft threshold in the primary contrasts were close to the analytic chance expectation, we used a more stringent selection criterion. Under the 4-versus-4 analytic null model, the probability of exceeding |*d*| ≥ 3 is approximately 0.54%, corresponding to an expected 0.72 selected pairs among the 133 packing contacts. We combined this effect-size threshold with a minimum absolute occupancy difference of |*Δocc*| ≥ 0.15 to retain contacts showing both large, standardized differences and substantial occupancy shifts. Table S4 reports the sensitivity of the selection to alternative thresholds, including the numbers of selected contacts, their permutation-derived chance expectations, and the retention of the highlighted contacts.

As a methodological caveat, the internal 2-versus-2 within-system control (Figure 2c,d) shows a heavier |*d*| tail than the primary 4-versus-4 design. The analytic null predicts 50.8% exceedance at |*d*| ≥ 0.8 for 2-versus-2, compared with 30.1% for 4-versus-4. The same numerical cutoff is therefore less stringent for the 2-versus-2 small-sample null, meaning its higher hit counts cannot be benchmarked directly against the primary contrasts.

### 2.4. Packing Contacts at TM1/TM6

Applying the hard filter allowed us to isolate specific packing differences. The WT–A305T contrast retained only the Gly59–Leu306 packing contact. The A305T–A346S contrast retained three pairs: Gly297–Ser302 (|*d*| = 6.43, *Δocc* = 0.75), Val58–Ala61 (|*d*| = 4.58, |*Δocc*| = 0.76), and Gly59–Leu306 (|*d*| = 3.28, |*Δocc*| = 0.48). No differential packing contacts in the WT–R211C contrast survived the filter.

Spatial proximity to the mutation site is insufficient to pass the hard filter alone. For example, the Gly301–Ala305 pair on the TM6 turn directly adjacent to the A305 site shifts occupancy by 0.73 in the A305T–A346S contrast, but its |*d*| value of 2.76 falls short of the threshold. In the WT–A305T contrast, the same pair yields *Δocc* = −0.45 and |*d*| = 1.11.

All three robust hits locate to the discontinuous TM1 and TM6 segments adjacent to the substrate site (Figure 3d): Gly297–Ser302 on the TM6 turn, Val58–Ala61 on TM1, and Gly59–Leu306 acting as an interhelical bridge. For Gly297–Ser302 and Val58–Ala61, wild-type occupancy lies intermediately between the A305T and A346S states (Figure 3a–c). This demonstrates that the two substitutions drive the local motif in opposite structural directions, rather than inducing a shared drift from the wild type. Fixed reference frames confirm that these packing shifts reflect genuine spatial distance changes rather than viewing artifacts.

To assess systemic displacement, we calculated occupancy-shift vectors by subtracting wild-type occupancies from those of A305T and A346S. These vectors exhibit a weak but statistically robust anti-correlation across the packing panel (*r* = −0.16, *r*^2^ = 0.026) (Figure 4a). Under the shared-wild-type permutation null, this anti-correlation is highly significant (*p* = 0.0038), whereas R211C contrasts show no such pattern (Figure 4b). Consequently, these variants exert distinct, opposing mechanical displacements at the TM1–TM6 interface.

We present the time courses for Gly297–Ser302 and Val58–Ala61 in Figure S4. We focus on the Gly59–Leu306 contact in the main text (Figure 5) because it forms an interhelical bridge between TM1 and TM6, distinguishes the two variants from the wild type and from each other, and cleanly separates the systems based on whole-window occupancy (A305T − WT *Δocc* = −0.58). In three of the four A305T replicates, this contact permanently breaks at 471, 548, and 588 ns. Conversely, the contact remains stable in three of the four wild-type replicates, breaking only near 738 ns in replicate 3.

Structurally aligned snapshots demonstrate that the Gly59–Leu306 heavy-atom distance in A305T (replicate 3) increases from 3.9 Å before the transition to 6.5 Å afterward (Figure 5a), crossing the interaction threshold and confirming the loss of the contact. In the corresponding wild-type state, this distance remains stable at 3.9 Å. Throughout these shifts, the TM1 and TM6 backbone traces maintain their original fold and relative orientation. This demonstrates that the contact loss results from a localized side-chain rearrangement rather than a global conformational change in the helix bundle.

### 2.5. Hydrogen Bonds Mark Substitutions Locally

The typed contact panel (*N* = 679: 645 hydrogen-bond pairs and 34 salt-bridge pairs, both filtered at mean occupancy ≥ 0.01 across all 16 replicates) used the same dual filter. Soft counts track chance (~205 expected, naive analytic). The naive analytic hard-threshold figure (~3.7 per comparison) makes the same normality assumption already shown to overstate the true floor for packing: the exact permutation null over all 35 distinct 4|4 relabelings, computed separately for the 645 hydrogen-bond pairs and the 34 salt-bridge pairs from per-replicate occupancy (Table S3), gives a dual-filter expectation of 1.27 hydrogen-bond hard hits per contrast (7.6 over six contrasts) and 0.076 salt-bridge hard hits per contrast (0.46 over six). Against that floor, the 21 observed hydrogen-bond hard hits are far above chance (Poisson *P*(*X* ≥ 21 | *λ* = 7.6) = 4.6 × 10^−5^), while the observed zero salt-bridge hard hits remain consistent with chance (P(*X* ≥ 1 | *λ* = 0.46) = 0.37). All 21 hard hits are hydrogen bonds; no salt bridge clears both thresholds. Informative contrasts appear for each variant versus wild type: four hard H-bonds for R211C, four for A346S and two for A305T.

When mapped onto the same annotation, the 21 hard-filtered contrast hits (|*d*| ≥ 3 and |*Δocc*| ≥ 0.15) resolve to 12 distinct residue pairs. Three of these pairs contain a substituted side chain, distributed across different functional regions.

First, the hydrogen bond between the carbonyl oxygen of Met197 and the guanidinium NH2–HH21 group of Arg211 is located in EL2. This loop contains the conserved Cys164–Cys173 disulfide bond and N-glycosylation sites (Asn176, Asn181, and Asn184) and shifts its proximity to EL4 in a conformation-dependent manner during the transport cycle [36]. Second, Gly112–Thr305 positions are on the cytoplasmic face of the unwound TM6 segment, one helical turn away from Ser295 and three residues from Ser302. Third, Ser346–Arg351 lies within EL4, where contacts with TM1 help close the extracellular pathway in the inward-facing conformation of GAT-1 [25] (Figure 6a–d).

### 2.6. Salt Bridges: Hard Count Zero

For the 34 evaluated salt bridges, the number of pairs passing the soft threshold approached the chance expectation of approximately 10. Conversely, no pairs survived the stringent filter in any contrast, including WT–R211C. Several pairs exhibited large |*d*| values driven by negligible shifts in fractional occupancy. The Lys278–Glu283 pair represents the most pronounced example, reaching a |*d*| of 7.7 alongside a minimal occupancy change (|*Δocc*| ≈ 0.07), thereby failing to satisfy the |*Δocc*| ≥ 0.15 criterion. At this sample size, a high |*d*| metric alone is insufficient to substantiate a robust salt-bridge rearrangement.

The pairs with the largest occupancy range across systems (Lys117–Glu411, Lys123–Glu575, and Lys117–Glu410) still miss |*d*| ≥ 3 on replicate scatter. Rolling occupancy shows salt bridges fluctuating on the microsecond timescale in mutants and wild type alike. Method 1 (replicate averages) remains empty after filtering; Method 2 (frame pooling) is not used as a second discovery engine.

Although the R211C substitution is predicted to disrupt Arg211-mediated salt bridges, the Arg211−Asp171 and Arg211–Glu165 pairs demonstrate near-zero occupancy in the wild-type state. Consequently, their absence in the mutant does not register as a structural loss under the stringent filter. The minimum distance between wild-type Arg211 and Asp171 is approximately 6.6 Å, strictly outside the 4.0 Å interaction cutoff. The sole differential polar signal retained for R211C is the Met197–Arg211 hydrogen bond.

### 2.7. The Extracellular Gate Remains Closed on This Timescale

The time-median constriction radii remain below the radius of a dehydrated sodium ion across all sixteen trajectories (0.34–0.74 Å versus 0.95 Å; Figure S3). Unpaired Cohen’s *d* values for the gate bottlenecks relative to the wild type are −0.28 (R211C), +0.45 (A305T), and +0.05 (A346S). None of these differences satisfy even the soft threshold of |*d*| ≥ 0.8, and they fall strictly below the stringent |*d*| ≥ 3 cutoff. Consequently, the observed TM1/TM6 packing modifications and local hydrogen-bond alterations represent localized structural repacking within a stable inward-open conformation with a closed extracellular gate, rather than a transition toward a conducting state.

### 2.8. Per-Replicate Dynamic Networks Reveal Variant-Specific Reorganization of Ligand-Binding Regions

Per-replica gMI networks were constructed from 1601 frames for each of the 16 trajectories using a common set of 10,102 candidate residue pairs. Network density varied across replicas and cutoffs, but the use of independent replica networks made it possible to distinguish recurrent residue-level changes from features specific to a single trajectory. The difference between WT and a variant (median MAE 0.070–0.081, correlation 0.771–0.796) was within the range seen between replicas of one system (MAE 0.067–0.093, correlation 0.759–0.811; Table S5). We therefore compared individual residues and routes and did not compare the mutant networks as a whole. gMI measures statistical dependence between residue fluctuations; the shortest path in the resulting graph is therefore a statistical construct and has limited predictive strength for signal transmission or transport efficiency.

The consensus centrality analysis identified a recurrent set of binding-site and gate-associated residues whose network roles changed across variants. Tyr140, Phe294, and Ser302 recurrently lost betweenness across mutant backgrounds, whereas Asp451 and Ser456 recurrently lost centrality, primarily in node strength (Figure 7). Centrality gains were variant-specific: R211C increased centrality at Gly65 and Thr400; A305T shifted centrality toward Tyr60, Ala61, and adjacent TM1 residues; and A346S increased centrality at Gly65 and Thr400, together with Ile338/Val342 and the 65–75 corridor. Node-strength results followed the same spatial pattern, with the largest reproducible shifts clustered around the substrate-binding core, the extracellular gate, and the TM1/TM6–EL4 interface.

Edge-level analysis identified Gly297–Ser456 as the most reproducible cross-variant loss of strong dynamic coupling. At gMI ≥ 0.45, this edge was retained in all four WT networks but fell below the inclusion threshold in all four replicas of R211C, A305T, and A346S. Its median gMI decreased from 0.497 in WT to 0.364 in R211C, 0.415 in A305T, and 0.424 in A346S; in R211C, the edge was absent from all four networks even at gMI ≥ 0.40. Variant-specific losses included the Gly297–Ala455 edge in all four R211C networks (gMI ≥ 0.40) and the Phe294–Asp451 edge in all four A346S networks (gMI ≥ 0.45). At the 0.45 cutoff, the R211C network also lost a broader cluster of edges between residues 282–298 and 455–460. Because the raw gMI values remained non-zero and absolute edge loss did not persist at the 0.35 cutoff, these findings represent the attenuation of high-confidence couplings from the thresholded network rather than complete structural disconnection.

R211C produced a consistent increase in communication-path length from position 211 to both ligand-relevant panels. Eight S1 targets showed robustly longer paths, and none showed shorter paths; the affected targets included Ala61, Gly63, Tyr140, Phe294, Ser295, Tyr296, Gly297, and Ser396. The largest changes occurred for the Phe294–Ser295–Tyr296–Gly297 region: across the three thresholds, the mutant-minus-WT path-length difference ranged from +0.273 to +0.432 for Phe294, +0.288 to +0.379 for Ser295, +0.261 to +0.352 for Tyr296, and +0.196 to +0.342 for Gly297. Five targets in the extended tiagabine-recognition panel also showed longer paths, including Phe294, Tyr296, Ala358, Tyr140, and Ile143. Exact routes varied among replicas, but weakening of the Leu218–Tyr140 relay was retained across the cutoff series (ΔgMI_median_ = −0.061). These path changes coincided with reduced betweenness or strength at Tyr140, Phe294, Tyr296, Gly297, and Ser302.

A305T divided the binding region into oppositely affected branches. Within the S1 panel, paths to Tyr60, Ala61, Gly63, and Gly65 were robustly shorter, whereas paths to Tyr140, Ser396, and Ala455 were longer. The shorter branch was organized around a recurrent Thr305–Tyr60 relay (ΔgMI_median_ = +0.099), followed by strengthened Tyr60–Gly65 (+0.167), Tyr60–Ala61 (+0.053), and Tyr60–Gly63 (+0.052) edges. In the vestibule/gate panel, paths to Trp68 and Ala358 were shorter, whereas those to Tyr140 and Ile143 were longer. This directional split was also present in the centrality analysis, where Tyr60 and Ala61 gained betweenness and strength, while Tyr140, Phe294, Ser302, Ser396, and Asp451 lost network prominence.

A346S generated the broadest increase in predicted communication efficiency. Eleven of thirteen S1 targets had robustly shorter paths, and none had longer paths; six of eight targets in the extended tiagabine-recognition panel were likewise reached by shorter paths. The largest S1 changes involved Tyr60 (ΔL ∈ [−0.371;−0.347]), Ser295 (−0.304 at all cutoffs), Tyr296 (−0.298), Phe294 (ΔL ∈ [−0.371;−0.347]), Ala61 (−0.269), and Gly297 (−0.250). A reproducible route from the substituted site to the S1 region was formed by Ser346–Phe339 (median ΔgMI = +0.103), Phe339–Phe70 (+0.120), and the terminal Phe70–Phe294 (+0.081) or Phe70–Ser295 (+0.092) relays. The vestibule network additionally contained strengthened Ile338–Trp68 (+0.154) and Ser346–Ala358 (+0.096) edges. Consistent centrality and strength gains at Gly65, Trp68, Pro71, Cys74, Val337, Ile338, Val342, and Thr400 connected this route to the TM1/EL4 side of the ligand-binding region.

## 3. Discussion

### 3.1. The Effects of the Dual Cohen’s d/Occupancy Filter

At *n* = 4 per system, a conventional ∣d∣ ≥ 0.8 threshold alone was not sufficiently selective: the analytic and permutation controls showed that many such calls can arise under replicate relabeling. Requiring both a large, standardized difference and a minimum absolute occupancy shift reduced the output to a small set of interaction-class-specific contacts. The resulting packing and hydrogen-bond signals exceeded their respective permutation-derived chance floors, whereas salt bridges did not. Because these layers did not converge on a common contact network, they are interpreted separately rather than combined into a single structural score.

### 3.2. Two Distinct Variant Phenotypes

R211C and the A305T/A346S pair leave different fingerprints. Loss of Arg211 invites a salt-bridge reading, yet the candidate Arg211···Asp/Glu pairs are already empty in wild type (~6.6 Å versus the 4.0 Å cut). What survives filtering is loss of Met197–Arg211/Cys211 (|*d*| = 3.76, *Δocc* = 0.39; Figure 6a)—local polar rearrangement at EL2, not electrostatic-network collapse.

A305T and A346S instead share a weak, significant negative shift–shift correlation at the same TM1/TM6 packing motif (*r* = −0.16, *r*^2^ ≈ 0.026). Three packing pairs clear the hard filter between them; wild-type occupancy for the intrahelical pairs sits between the mutants; and the correlation is extreme under the shared-WT permutation null (*p* = 0.0038; Figure 4), unlike either R211C contrast. The time-resolved behavior of the Gly59–Leu306 contact makes it the clearest structural evidence. The bridge irreversibly dissociates within the production window in three of the four A305T trajectories but largely persists in the wild-type simulations (Figure 5). The hard threshold eliminates all salt bridges, including contacts within the R211C structure. Applying the |*Δocc*| co-filter makes this negative result interpretable. The Lys278–Glu283 interaction illustrates this mechanism, reaching a |*d*| metric of approximately 7.7 alongside an occupancy shift of *Δocc* ≈ 0.07.

### 3.3. Local Network Reorganization Converges on Ligand-Binding Regions

Network analysis links local contact changes to global transporter dynamics. Across all three variants, whole-network gMI divergence remained within the range defined by independent replicas, in agreement with a closed extracellular gate. The substitutions therefore act primarily by redistributing communication through selected residue networks rather than by producing generalized destabilization of the inward-open GAT-1 fold.

For R211C, the mutation-proximal loss of the Met197–Arg211 hydrogen bond was accompanied by longer communication paths to both ligand-relevant panels. The largest increases terminated at the Phe294–Ser295–Tyr296–Gly297 segment located in the unwound central segment of TM6, where the regular α-helical structure is interrupted to form one wall of the primary S1 binding site. Within this segment, Phe294 forms part of the extracellular roof of the pocket and its backbone carbonyl contacts GABA, whereas Ser295 participates in Na1 and Cl^−^ coordination; Tyr296 and Gly297 complete the substrate-facing TM6 surface. Tyr140, on the opposing TM3 side, directly recognizes GABA and participates with Phe294 in closure of the extracellular gate [19,25]. Weakening of the Leu218–Tyr140 relay and longer paths to the TM6 segment demonstrate that the distal EL2–TM4 perturbation uncouples from the residues coordinating substrate and ion occupancy with extracellular-gate rearrangement.

A305T redistributed intrapocket couplings instead of weakening the network uniformly. Ala305 lies at the TM6a–TM6b break next to the S1 site; its replacement by threonine disrupted the Gly59–Leu306 TM1/TM6 packing contact and introduced a local Thr305–Gly112 hydrogen bond. In parallel, stronger Thr305–Tyr60–Ala61/Gly63/Gly65 communication coupled the mutated TM6 turn more tightly to the TM1 side of S1. This branch is functionally positioned because Tyr60 contributes to substrate recognition, the Ala61 backbone participates in Na1 coordination, and Gly63/Gly65 line the pocket [19,25]. By contrast, longer paths to Tyr140 and Ser396 weakened communication with the opposing TM3/TM8 ligand-contacting surface, while changes involving Ile143 and Ala455 extended this loss toward the vestibule and TM10 gate side. A305T introduces an asymmetry into the binding-site network. The mutation reinforces the TM1-facing branch while decoupling the opposing substrate-recognition and gating elements, which can desynchronize local substrate/Na^+^ coordination from gate motion even without collapse of the pocket.

A346S instead reinforced dynamic coupling. Position 346 lies in EL4 between TM7 and TM8, and the mutation-proximal Ser346–Arg351 hydrogen bond stabilizes this local loop environment. The recurrent Ser346–Phe339–Phe70–Phe294/Ser295 route connects EL4 to the extracellular end of TM1 and then to the TM6 substrate/ion-binding segment; the Ile338–Trp68 and Ser346–Ala358 branches additionally connect EL4 to the extracellular vestibule. The assignment of Phe294/Ser295 to the substrate/ion-binding TM6 segment and of Trp68 and the surrounding TM1 region to the extracellular vestibule is supported by the resolved GAT1 substrate- and inhibitor-bound structures [19,24]. Shorter paths to most S1 and vestibule targets indicate increased coordination among EL4, the outer vestibule, the extracellular gate, and the central pocket in the inward-open ensemble. Because alternating access requires these elements to change partners during state transitions, stronger coupling does not equal improved transport. Instead, it biases the inward-open communication pattern and reduces the flexibility needed to proceed through the transport cycle.

The recurrent weakening of Gly297–Ser456 represents a shared structural defect across all variants. Gly297 belongs to the non-helical TM6 segment lining S1, whereas Ser456 lies on TM10 and participates in the hydrogen-bond network formed with Arg69, Gln291, and Asp451 during extracellular-gate closure [19]. Loss of this high-confidence edge from every mutant network at the strict cutoff therefore indicates weakened dynamic coordination between the substrate-binding TM6 segment and the TM10 side of the extracellular gate. The broader loss of edges between residues 282–298 and 455–460 in R211C suggests that this pocket-to-gate decoupling is most extensive in that variant. However, the non-zero raw gMI values and recovery of these edges at the lowest cutoff preclude interpreting their thresholded loss as complete physical separation.

All three variants disrupt the substrate pocket–extracellular gate axis via distinct dynamic mechanisms. R211C causes distal decoupling, reducing signal propagation from EL2–TM4 to the TM6 substrate/ion-binding segment and Tyr140 gate region. A305T creates an asymmetric S1 network that favors the TM1-facing Tyr60 branch while weakening communication with the opposing TM3/TM8 and TM10 gate side. A346S reinforces an EL4-to-TM1/TM6 corridor, potentially over-stabilizing an inward-open coupling pattern that must subsequently be released for alternating access. These mechanisms yield specific testable predictions, including impaired long-range signal propagation for R211C, mismatched substrate/ion-site and gate coordination for A305T, and excessive state-specific coordination for A346S. Resolving these functional consequences requires ligand- and ion-bound simulations across additional conformational states.

### 3.4. Linking Structural Dynamics to Variant Severity and Functional Impairment

Comparative dynamic analysis demonstrates that these variants disrupt GAT-1 via distinct structural pathways, as opposed to a uniform global destabilization. The strongest evidence concerns A305T, which directly reorganizes the discontinuous TM1–TM6 interface adjacent to the substrate- and ion-binding core. The Gly59–Leu306 contact, the only selected bridge between TM1 and TM6, decreased in fractional occupancy from 0.80 in the wild type to 0.23 in A305T. Following contact loss at 471, 548, and 588 ns in three of four replicates, this bridge did not reform during the remainder of the simulation. This bridge links Gly59, which contributes to Na2 coordination [25], to Leu306 immediately adjacent to residue 305. In parallel, the Gly297–Ser302 contact on the GABA/tiagabine-facing side of TM6 increased in occupancy from 0.54 to 0.95, whereas the Val58–Ala61 contact in the sodium-site region decreased from 0.60 to 0.15. A newly formed Gly112–Thr305 hydrogen bond further tethered IL1 to the cytoplasmic face of the unwound TM6 segment. Thus, A305T simultaneously removes a TM1–TM6 bridge required for transporter reset, alters packing on the substrate-facing side of TM6, and introduces a new cytoplasmic tether that likely increases the energetic cost of inner-gate rearrangement. These changes may impair GABA binding and ion–substrate coupling, thereby increasing the energy barrier for transition to the outward-facing state; they do not by themselves distinguish among these possibilities. The experimentally reported severe uptake loss despite wild-type-like surface localization [18], together with the high AlphaMissense score of 0.976, is consistent with this simulation-derived hypothesis but does not validate the specific structural rearrangements observed here.

R211C induces a qualitatively different and less direct structural perturbation. Its only mutation-proximal alteration surviving the stringent filter is the loss of the Met197–Arg211 interaction in EL2, a region implicated in folding and membrane delivery [36]. In contrast to A305T, the central TM1/TM6 substrate- and ion-binding architecture in R211C remains largely wild-type-like. Instead, the primary systemic consequence is the distal decoupling of the EL2–TM4 segment from the TM6/S1 core and the extracellular-gate elements.

The available functional evidence aligns with this milder structural phenotype. In the [^3^H]-GABA uptake assay, R211C showed an intermediate loss of function, retaining 24.7% of wild-type activity [18]. Together with an intermediate AlphaMissense score of 0.723, this finding supports partial functional impairment, in contrast to the severe, direct transport-cycle defect observed for A305T.

A346S exhibits the weakest evidence for pathogenicity among the three variants, despite inducing the most extensive set of indirect structural perturbations. The Gly59–Leu306 bridge retains wild-type-like fractional occupancy (0.70 versus 0.80), and the local TM1/TM6 shifts proceed in the opposite direction compared to A305T. The pronounced mutation-proximal Ser346–Arg351 occupancy gain directly results from the introduction of the serine side chain; therefore, it does not constitute independent evidence of global transporter dysfunction. Excluding this direct local interaction, the remaining structural consequence consists of rigidified EL4 coupling and a cytoplasmic-gate shift. These alterations are compatible with modified state-specific gating but could equally result in a mild or negligible functional deficit. This interpretation aligns with the benign AlphaMissense score of 0.113 [26], although direct functional uptake assays are currently lacking. Consequently, we predict that A346S retains greater transport activity than A305T and potentially matches or exceeds that of R211C. This relative ranking represents a computational prediction that awaits experimental validation.

Current functional and structural data establish A305T as more disruptive than R211C, while structural modeling predicts A346S to be the least severe of the three variants. The combined results link these variants to two experimentally anchored mechanisms, identifying direct TM1/TM6–S1/ion-site reorganization for A305T and distal EL2–TM4-to-pocket/gate decoupling for R211C. Direct measurements of GABA uptake, substrate affinity, ion dependence, surface expression, and conformational kinetics for A346S will be required to complete this ranking.

### 3.5. Limits of the Inward-Open Apo GAT-1 Model

The extracellular gate remains closed throughout the simulations, with a HOLE2 time-median constriction of 0.34–0.74 Å across all 16 replicates compared to 0.95 Å for dehydrated Na^+^ and 1.40 Å for water (Figure S3). Consequently, all identified packing and hydrogen-bond alterations occur within a stable inward-open, gate-closed conformation. The analysis carries three methodological limitations. First, the structural models include TM12 but lack the distal C-terminal tail (residues 578–599).

Second, the simulations evaluate only the apo-state, excluding substrate- and ion-bound complexes. Consequently, the observed structural and dynamic changes support mechanistic hypotheses but cannot be interpreted as direct measurements of GABA flux, transport turnover, or ion–substrate coupling. Under the present setup and accessible timescales, equilibrium MD samples fluctuations around the initial inward-open state rather than reproducing complete alternating-access transitions and transmembrane transport events.

Third, the predefined region of interest for packing analysis excludes residue pairs near R211 or A346, leaving local packing dynamics at these mutation sites untested. Given these constraints, the filtered contacts provide structural annotations to guide future experimental work rather than establishing direct pathogenic mechanisms or providing evidence for ACMG/AMP classification and therapeutic prioritization.

## 4. Materials and Methods

### 4.1. Structural Model and Simulated Systems

The PDB-deposited structure of inward-open apo cryo-EM human GAT-1 7Y7V with 2.2 Å resolution was used in this study [19]. The model spans residues 46–577. The distal cytoplasmic N- and C-tails are missing in 7Y7V and therefore absent from the trajectories. Residue numbers follow UniProt numbering. Three missense models (R211C, A305T, and A346S) were made by *in silico* mutagenesis in PyMOL.

Each protein was oriented and positioned in the membrane using the PPM 2.0 algorithm implemented in CHARMM-GUI Membrane Builder. Protein–membrane systems were then assembled using the replacement method in a mixed bilayer containing 48.2 mol% cholesterol (CHL1), 32.5 mol% 1,2-dilauroyl-sn-glycero-3-phosphoethanolamine (DLPE), 8.4 mol% 1,2-dilauroyl-sn-glycero-3-phosphocholine (DLPC), 6.0 mol% 1,2-dimyristoyl-sn-glycero-3-phosphoinositol (DMPI), 2.4 mol% N-tricosanoyl-sphingomyelin (23SM), and 2.4 mol% N-lignoceroyl-sphingomyelin (LSM). The system was solvated in TIP3P water and neutralized at 150 mM KCl.

Each system underwent the standard CHARMM-GUI energy-minimization and equilibration steps. Starting from a single equilibrated endpoint, four independent 1-µs production replicas were performed at 310.15 K and 1 bar. Independent stochastic trajectories were generated across replicas by the velocity-rescale thermostat.

### 4.2. Three Interaction Layers

The first 200 ns were excluded as an initial relaxation period, and a common 200–1000 ns window was used for subsequent analyses. This operational cutoff does not imply complete structural convergence, as small residual RMSD drifts persisted in some trajectories. Analyses were performed on protein-only trajectories sampled at 50 ps intervals, yielding 16,001 frames per replicate and 256,016 frames across sixteen replicates. Packing contacts, hydrogen bonds, and salt bridges were identified using the geometric criteria described below. A packing contact was counted in a frame when the minimum heavy-atom distance was ≤4.5 Å. Packing analyses used a fixed panel of 133 residue pairs spanning the GAT-1 functional core inner surface (residing in TM1, TM3, TM6, and IL4). Hydrogen bonds were identified using a fixed set of donor and acceptor heavy atoms spanning backbone and side-chain nitrogen, oxygen, and sulfur. Backbone donors were the amide nitrogen of every residue, and backbone acceptors were the carbonyl oxygen of every residue. Side-chain donors were Arg NE, NH1, and NH2, Lys NZ, Trp NE1, Asn ND2, Gln NE2, and His ND1 and NE2. His ND1 and NE2 were treated as donors throughout and as acceptors only on histidine, reflecting the imidazole ring’s tautomer-dependent protonation state. Side-chain acceptors were Asp OD1 and OD2, Glu OE1 and OE2, Asn OD1, and Gln OE1. Ser OG, Thr OG1, Tyr OH, and Cys SG were treated as both donor and acceptor. A hydrogen bond required a donor–acceptor heavy-atom distance ≤ 3.5 Å and a donor–hydrogen–acceptor angle ≥ 150°. Salt bridges were scored between the carboxylate oxygens of Asp/Glu and the cationic nitrogens of Arg/Lys when the minimum inter-atom distance was ≤4.0 Å. Residue pairs separated by fewer than four residues in sequence were excluded as trivial local contacts.

Polar features entered a typed set of 679 pairs (645 hydrogen bonds, 34 salt bridges) with mean occupancy ≥ 0.01 across systems; the 34 salt bridges were also analyzed alone. Replicate occupancy is the fraction of 200–1000 ns frames meeting the geometric cut. *Δoccupancy* for a contrast is the difference of the two system means of those replicate occupancies.

### 4.3. Effect Size, Soft and Hard Filters

For each contact, the four replicate occupancies per system were compared across six pairs: WT–R211C, WT–A305T, WT–A346S, R211C–A305T, R211C–A346S, and A305T–A346S. Unpaired Cohen’s *d* [37] was calculated using the pooled sample standard deviation (ddof = 1), alongside the absolute difference in mean occupancy. Contacts were first tabulated at the soft threshold |*d*| ≥ 0.8. Final hard selection required both |*d*| ≥ 3 and |*Δocc*| ≥ 0.15. Sensitivity to alternative cutoffs is reported in Table S4. To estimate the chance number of hard selections at *n* = 4 per system, the eight replicate occupancy vectors in each contrast were exhaustively reassigned across all 35 distinct 4|4 label allocations while preserving the complete contact vector of each replica. The dual filter was reapplied after each reassignment, and the mean number of retained contacts defined the permutation-derived chance floor.

Pearson correlations between global occupancy-shift vectors sharing a wild-type baseline (for example, A305T − WT versus A346S − WT) were evaluated at the level of the full shift vector across all contacts within a panel, not per individual contact, and were computed separately for each of the three feature panels—packing (133 pairs), hydrogen bonds (645 pairs), and salt bridges (34 pairs). Each vector holds one occupancy-shift value per pair in that panel, and the resulting correlation coefficient is a single summary statistic for that contrast pair within that panel, not a per-contact statistic. Because shared wild-type noise enters both vectors with the same sign, the null correlation between them is not zero; significance was therefore assessed against a replicate-label permutation null (20,000 permutations) that reshuffles replicate labels while keeping each replicate’s full occupancy vector intact, preserving the shared-baseline structure. This test was run once per contrast pair sharing a WT baseline, per panel—not once per contact.

### 4.4. Pore Radius

Pore radii were computed with HOLE2 [38] on the 200–1000 ns window, sampled every 2.5 ns (321 profiles per replicate; 5136 in total). Frames were superposed on one transmembrane-core reference so that a single axis and reaction coordinate serve all sixteen replicates. The coordinate was anchored on the conserved GAT-1 R69–D451 gate, closed in the reference (2.69 Å closest side-chain approach) and coincident with the global minimum of the median radius profile, which checks the axis independently of the trajectories.

### 4.5. Dynamic Residue Network Analysis

Dynamic residue networks were calculated independently for each of the four replicas of WT, R211C, A305T, and A346S. Analyses used the 200–1000 ns production window sampled every 0.5 ns, yielding 1601 frames per replica. Each of the 532 residues resolved in the simulated GAT-1 model was represented by its C_α_ atom. Generalized mutual information (gMI) was estimated for residue-pair coordinate fluctuations using a k-nearest-neighbor estimator with k = 5. The resulting normalized gMI values range from 0 to 1, with larger values indicating stronger dynamic coupling.

A common candidate-edge mask was used for all 16 replica networks. A residue pair entered the mask when its mean C_α_ separation was ≤12 Å in at least one replica, producing a union of 10,102 candidate pairs. Applying the same mask to every system ensured that between-system comparisons were not driven by different geometric preselection. Undirected weighted graphs were constructed at three gMI cutoffs (0.35, 0.40, and 0.45). Edge weight was the corresponding gMI value, and edge length was defined as *d_ij_* = 1 − gMI_ij_; strongly coupled residues therefore formed shorter network connections.

For each replica and cutoff, residue importance was quantified by normalized weighted betweenness centrality and node strength, defined as the sum of gMI weights incident on a residue. For each mutant, the median value across its four replicas was compared with the WT median. A directional change was considered replicate-consistent when at least three of four mutant replicas were above or below the WT median, and robust when the same direction was retained at all three gMI cutoffs.

Whole-network similarity was evaluated on the unthresholded gMI vectors of all 10,102 candidate pairs. Pairwise mean absolute error (MAE), root-mean-square difference, and Pearson correlation were calculated for WT–WT, mutant–mutant, and WT–mutant replica pairs, and the median of each comparison class was used to describe the relation between within-condition variability and between-condition divergence.

Local communication was evaluated by weighted shortest paths from each variant position to two prespecified ligand-relevant panels. The S1 substrate-binding panel comprised Tyr60, Ala61, Gly63, Gly65, Tyr140, Phe294, Ser295, Tyr296, Gly297, Leu300, Ser396, Thr400, and Ala455. The extended tiagabine-recognition vestibule/gate panel comprised Trp68, Arg69, Tyr140, Ile143, Phe294, Tyr296, Leu300, and Ala358. Path length was the sum of 1 − gMI over all constituent edges. Mutant-versus-WT path changes were classified using the same three-of-four-replica and three-cutoff consensus rule. Relay edges were retained when they occurred in the mutant shortest paths in at least three replicas at every cutoff; their raw gMI changes were then summarized relative to the WT replica median.

### 4.6. Software

Membrane systems were built in CHARMM-GUI v3.7 [39] with CHARMM36m [40] and TIP3P [41], then converted to GROMACS. Equilibration followed CHARMM-GUI step6; production used GROMACS 2025.2 [42]. Trajectory analysis used MDAnalysis 2.10.0 [43,44], NumPy 2.4.6 [45], SciPy 1.17.1 [46] and pandas 3.0.3 [47]. Figures: Matplotlib 3.10.9 [48]; atomic views: PyMOL 3.1.0 Open-Source (1bebc9f500), 24 March 2026 [49]; pore radii: HOLE 2.3.1 [38].

## 5. Conclusions

Molecular dynamics simulations demonstrate that the investigated missense variants do not cause global destabilization of GAT-1. The overall inward-open conformation and closed extracellular gate persist. None of the three substitutions destabilized GAT-1’s overall fold. Extended simulations confirmed that the transporter retains an inward-open conformation, maintaining a closed extracellular gate that effectively blocks sodium ion permeation. Because the structural perturbations are localized and variant-specific, we propose three candidate mechanisms. These remain predictive models inferred from contact occupancies, pending experimental functional validation.

Based on MD trajectory analysis, we predict that the A305T substitution may reorganize the TM1–TM6 interface near the substrate- and ion-binding site, rupturing the Gly59–Leu306 contact and generating an asymmetric interaction network within the central pocket. R211C may alter the protein indirectly, as the loss of the local Met197–Arg211 interaction in EL2 weakens its coupling to the substrate-binding pocket and the extracellular gate. The A346S variant can induce the mildest structural deviations, preserving the critical Gly59–Leu306 bridge but restricting structural mobility by strengthening EL4 interactions with TM1/TM6, potentially over-stabilizing the inward-open state.

As the present analysis is restricted primarily to a single structural state of the transporter, it cannot determine whether the identified structural disruptions persist across other stages of the transport cycle or how they influence the transitions between these states. Consequently, future research must systematically investigate multiple functional states of GAT-1. First, future studies should examine the binding of the physiological substrate GABA alongside pharmacologically active GAT-1 ligands. Testing tiagabine is specifically required, as this selective GABA reuptake inhibitor stabilizes the transporter’s inward-open conformation [19,24]. Comparing the WT and mutant variants via ensemble molecular docking followed by molecular dynamics simulations of the resulting complexes will determine how the mutations affect ligand binding and the stability of the resulting complexes within the central binding pocket.

Second, the GAT-1 conformational cycle itself requires explicit modeling. Targeted and enhanced molecular dynamics methods can simulate the transitions between the outward-open, occluded, and inward-open states, identifying the specific transport cycle stage that exhibits the most pronounced differences between the WT and mutant forms. This analysis will shift the focus from describing static local rearrangements to evaluating whether specific mutations alter the energy barriers governing the opening and closing of the intracellular and extracellular gates.

Third, evaluating the mutant variants in the presence of GABA and the coupled Na^+^ and Cl^−^ ions is essential. GABA binding in GAT-1 is intimately linked to a coordinated hydrogen-bonding network and ion coordination, with conformational rearrangements in TM1 serving as an integral component of the substrate and Na^+^ release mechanism [19]. Experimental evidence further corroborates the tight coupling of GABA transport to Na^+^, along with the functional necessity of Cl^−^ throughout the GAT-1 transport cycle [50]. Consequently, simulating the fully assembled transport complex will allow us to determine how the A305T, A346S, and R211C substitutions alter ion and GABA binding, and whether they disrupt the propagation of conformational changes to the gating elements.

Defining these variant-specific biophysical mechanisms provides a structural basis for interpreting SLC6A1 mutations and guiding the rational identification of compounds capable of stabilizing distinct transporter states or rescuing specific molecular defects. In this context, tiagabine should be considered primarily as a structurally and pharmacologically well-characterized reference ligand for GAT-1 rather than a targeted therapeutic agent for *SLC6A1*-associated conditions, as GAT-1 inhibition in *SLC6A1*-related neurodevelopmental disorders can exacerbate certain seizure types [15].

Ultimately, integrating structural modeling with genetic and functional datasets could complement genetic and functional evidence in future variant interpretation for precision psychiatry. Such a framework facilitates a transition from classifying variants solely by their primary sequence position toward identifying discrete molecular pathomechanisms, potentially guiding pharmacological strategies tailored to the specific mode of GAT-1 dysfunction across individual genetic variants.

## Figures and Tables

**Figure 1:**
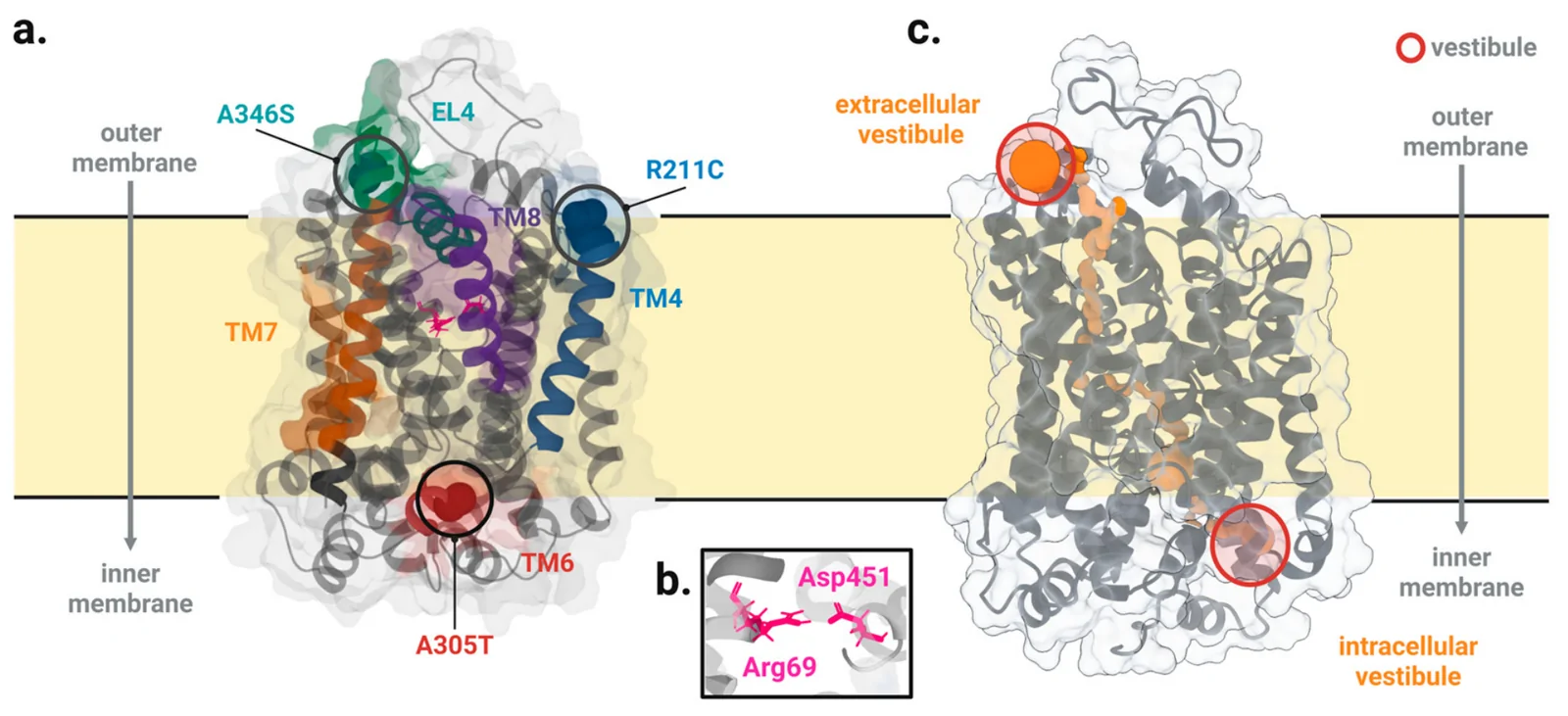
Structure of the simulated GAT-1 variants and permeation pathway. (**a**) Locations of R211C (blue), A305T (red), and A346S (teal). (**b**) The conserved Arg69–Asp451 pair at the TM1–TM10 interface, shown as a structural landmark of the extracellular gate. (**c**) HOLE2-derived permeation pathway in apo GAT-1 inward-open, extracellularly closed conformation; the extracellular and intracellular vestibules are indicated with red circles. The vicinity of Arg69–Asp451 corresponds to the narrow extracellular constriction, whereas the intracellular pathway remains accessible. Created in BioRender (2026) https://BioRender.com/86v8mge.

**Figure 2:**
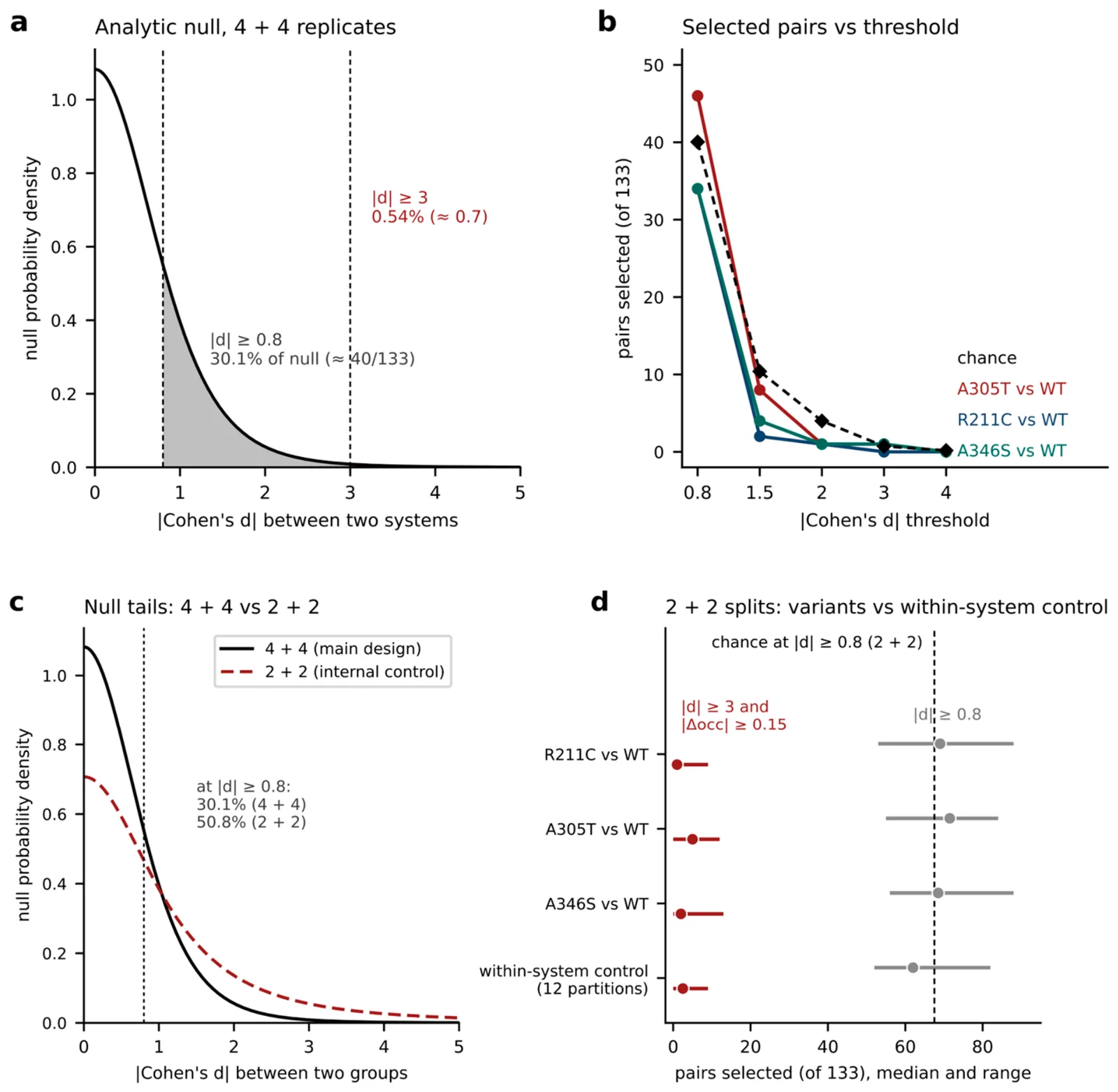
Calibration of contact-selection thresholds and within-system controls. (**a**) Analytical null distribution of |*d*| for the 4 + 4 replicate design. At |*d*| ≥ 0.8 and |*d*| ≥ 3, null exceedance probabilities are 30.1% and 0.54%, corresponding to approximately 40 and 0.7 of 133 contacts. (**b**) Packing contacts selected in variant–WT comparisons at increasing |*d*| thresholds; dashed line, analytical null expectation. (**c**) Null distributions for 4 + 4 and 2 + 2 designs, with exceedance probabilities at |*d*| ≥ 0.8 of 30.1% and 50.8%, respectively. (**d**) Contact counts in 2 + 2 variant–WT comparisons and 12 within-system control partitions using |*d*| ≥ 0.8 (grey) or |*d*| ≥ 3 and |*Δocc*| ≥ 0.15 (red). Points and bars show medians and ranges; dashed line, 2 + 2 null expectation at |*d*| ≥ 0.8. *Δocc*, difference in mean contact occupancy.

**Figure 3:**
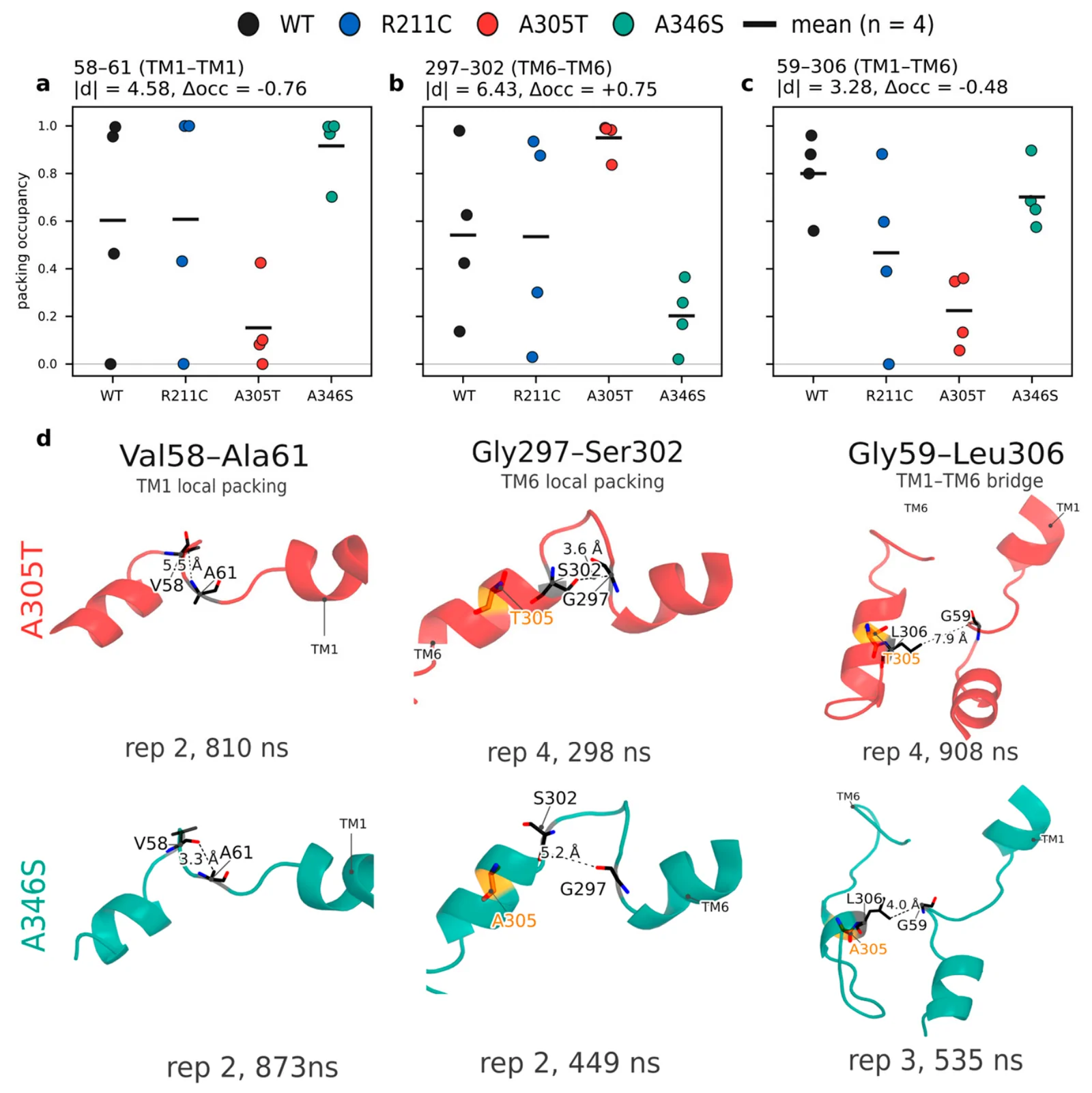
Packing hard hits. (**a**–**c**) Occupancy strips for Gly297–Ser302, Val58–Ala61 and Gly59–Leu306 across WT, R211C, A305T and A346S (*n* = 4). (**d**) Atomic views of the contacts in A305T (**top**) and A346S (**bottom**); dashed lines mark interatomic distances.

**Figure 4:**
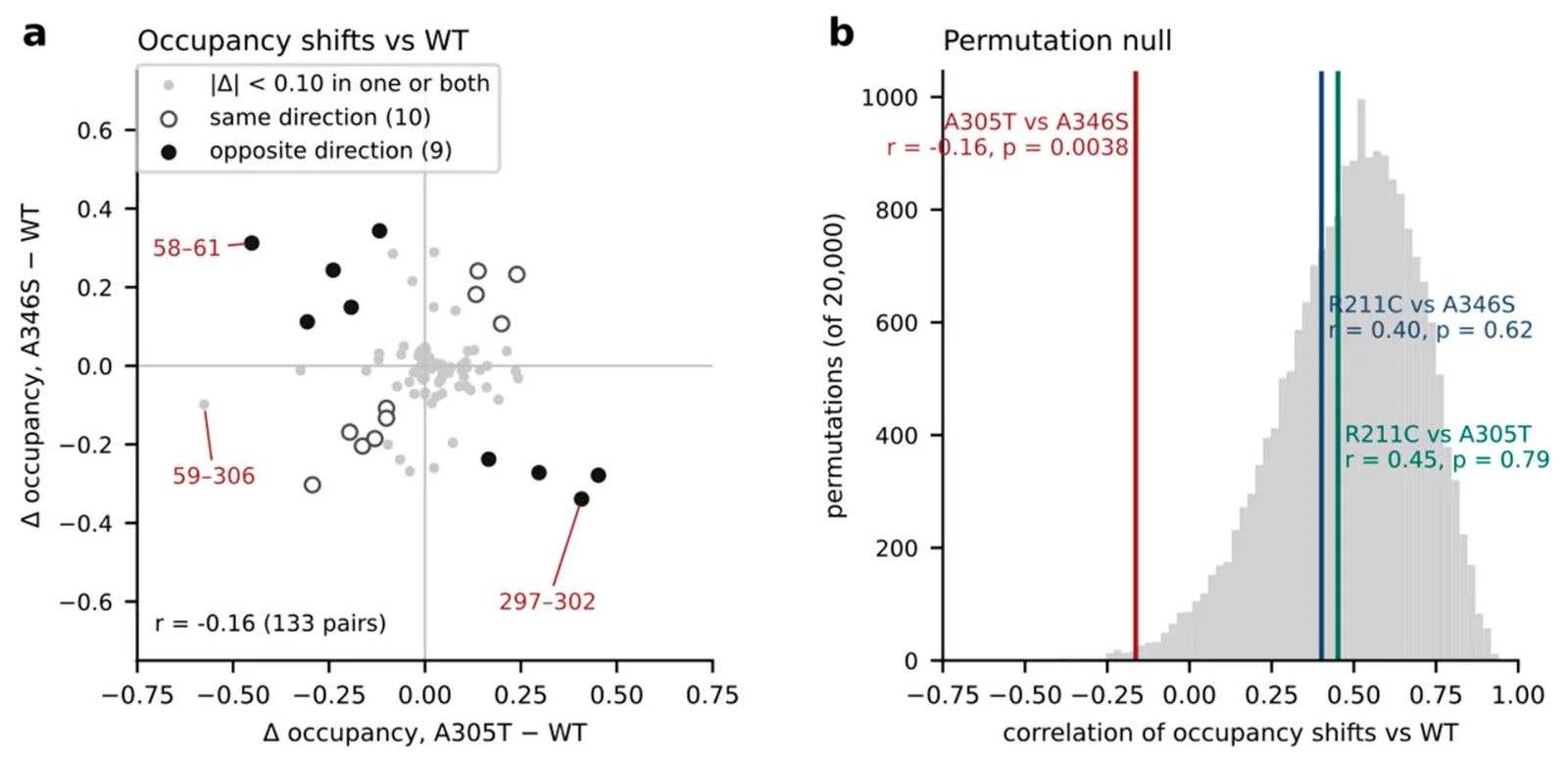
Direction of packing shifts. (**a**) A346S − WT versus A305T − WT occupancy shifts. (**b**) Observed correlations versus the shared-WT permutation null.

**Figure 5:**
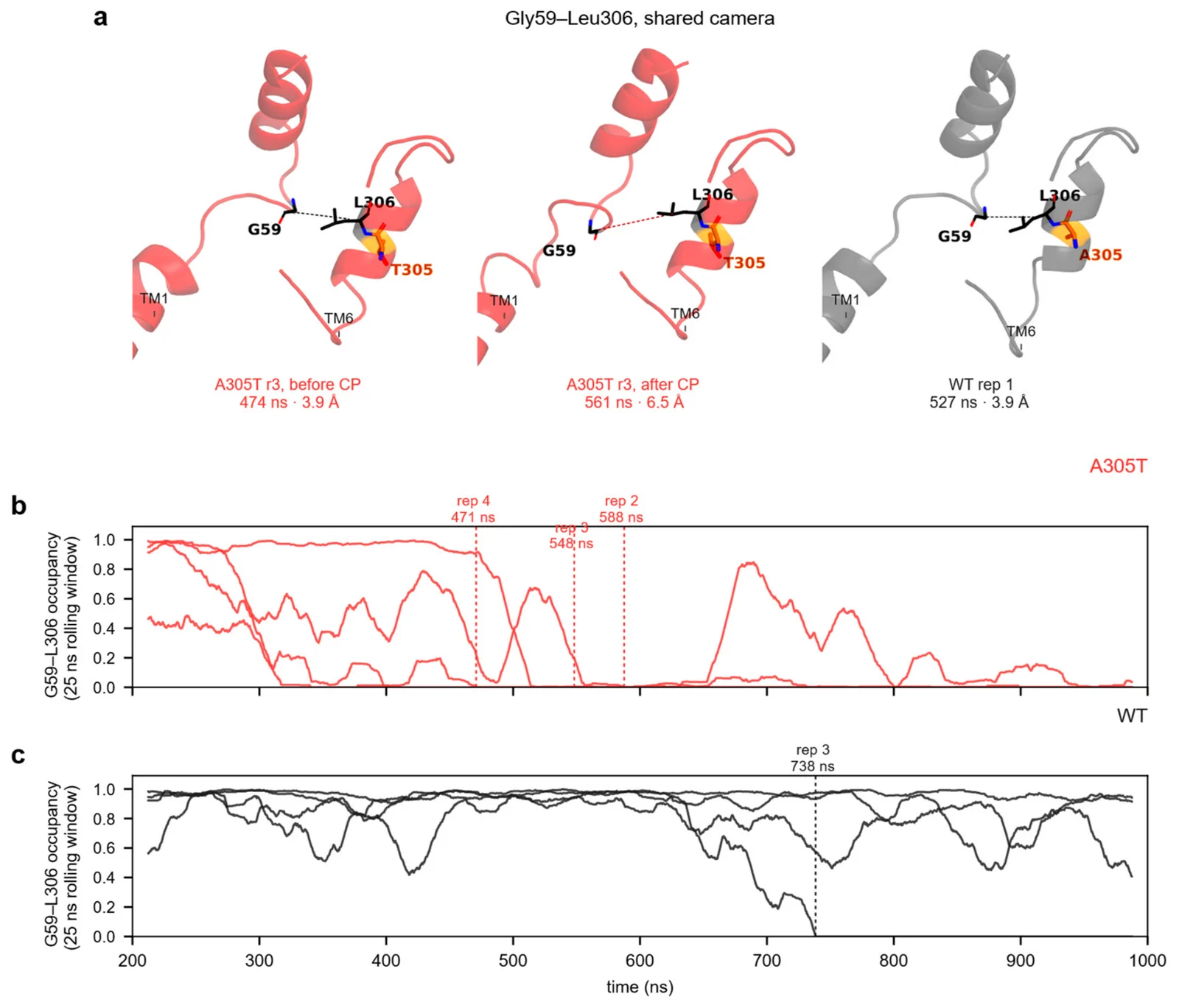
Gly59–Leu306 dynamics. (**a**) Shared-camera frames (A305T before CP, A305T after CP, WT; CP, change point of contact loss). (**b**) A305T rolling occupancy. (**c**) WT rolling occupancy.

**Figure 6:**
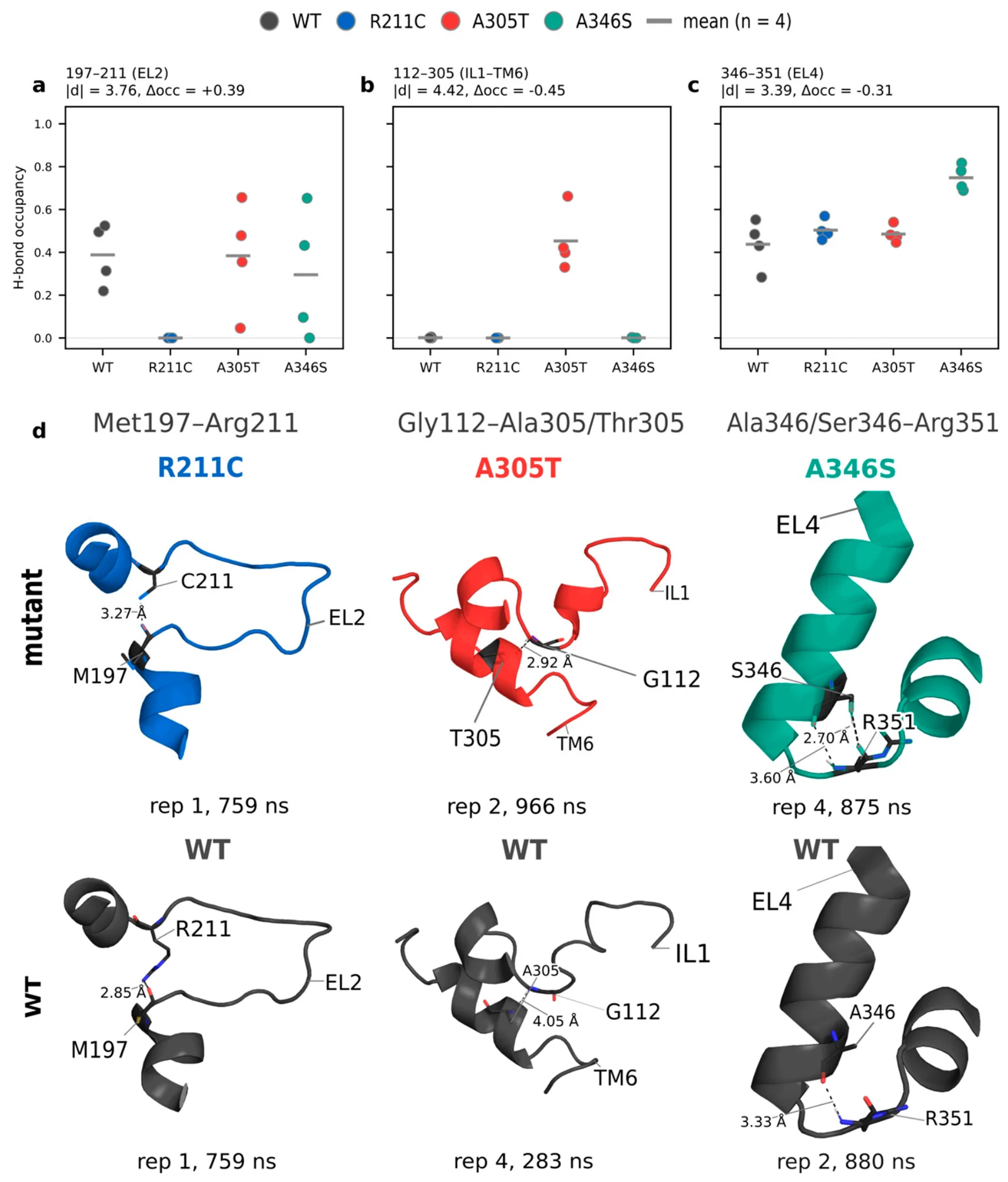
Mutation-proximal hard hydrogen bonds. (**a**–**c**) Occupancy of 197−211, 112−305 and 346−351. (**d**) Atomic views of the three variant sites (top row, mutant; bottom row, WT).

**Figure 7:**
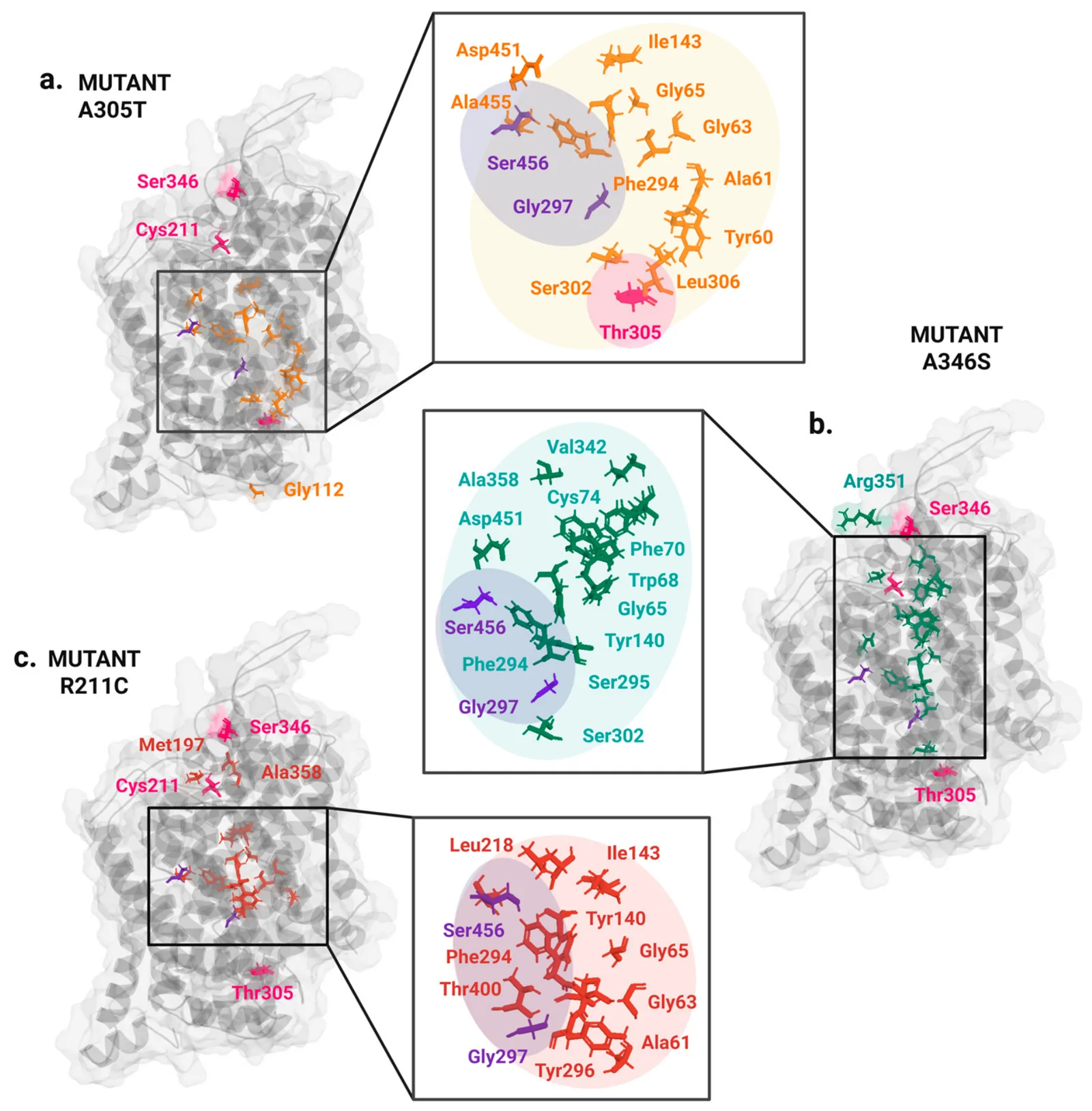
Variant-specific redistribution of local interactions and dynamic communication in GAT-1. Residues identified by the hard-contact and gMI network analyses are mapped onto the inward-open GAT-1 structure for (**a**) A305T, (**b**) A346S, and (**c**) R211C. Enlarged views show spatial clusters connecting the variant sites with the substrate-binding pocket and extracellular-gate region. Residues associated with A305T, A346S, and R211C are shown in orange, teal, and red, respectively; magenta marks the three variant positions. Gly297 and Ser456 are shown in purple to indicate the recurrent cross-variant weakening of their high-confidence dynamic coupling. The protein is shown in gray, and shaded regions group spatially related residues. Created in BioRender (2026): https://BioRender.com/d5vgvrj.

## Data Availability

GROMACS equilibration and production parameters (CHARMM-GUI step6.3–step6.6 *.mdp and production mdout_v2.mdp; GROMACS 2025.2, CHARMM36m) are available from the corresponding author. The 16 × 1 µs trajectories are available from the corresponding author upon reasonable request.

## Supplementary Materials

The following supporting information can be downloaded at: https://www.mdpi.com/article/10.3390/ijms27219793/s1.
