# Supplementary material for: Molecular Dynamics Reveals Structural Consequences of Schizophrenia-Associated SLC6A1 Variants
Source: Int J Mol Sci. 2026 Oct 31;27(21):9793. doi: 10.3390/ijms27219793 (PMC13763465; doi:10.3390/ijms27219793)
Supplement: Supplementary file 1 [file ijms-27-09793-s001.zip › TableS4.pdf]

Table S4. Sensitivity of the dual selection filter to its thresholds. For each combination of |d| and |Δocc| cutoffs, the number of interactions selected across the six pairwise system contrasts is compared with the number expected by chance, defined as the mean over all 70 distinct 4|4 relabellings of the eight replicate occupancy vectors of each contrast. Highlighted: the thresholds used in the manuscript.

|d| cutoff	|Δocc| cutoff	Selected	Expected by chance	Enrichment	Retained, of the 7 featured	Retained, of the 25 selected at used thresholds
2.0	0.10	83	42.5	1.95	7	25
2.0	0.15	67	32.6	2.06	7	25
2.0	0.20	57	25.8	2.21	7	23
2.0	0.25	48	21.3	2.26	7	18
2.5	0.10	46	20.0	2.30	7	25
2.5	0.15	36	16.1	2.24	7	25
2.5	0.20	31	13.3	2.32	7	23
2.5	0.25	24	11.2	2.14	7	18
3.0	0.10	32	10.8	2.97	7	25
**3.0**	**0.15**	**25**	**9.1**	**2.76**	**7**	**25**
3.0	0.20	23	7.5	3.06	7	23
3.0	0.25	18	6.5	2.78	7	18
3.5	0.10	20	6.2	3.21	5	16
3.5	0.15	16	5.4	2.96	5	16
3.5	0.20	15	4.5	3.37	5	15
3.5	0.25	12	3.9	3.09	5	12
4.0	0.10	15	3.9	3.83	3	11
4.0	0.15	11	3.5	3.18	3	11
4.0	0.20	11	3.0	3.63	3	11
4.0	0.25	8	2.6	3.11	3	8

The 7 featured interactions are the selections shown with atomic panels (Gly297–Ser302, Val58–Ala61 and Gly59–Leu306 in A305T vs A346S; Gly59–Leu306 and Gly112–Thr305 in WT vs A305T; Met197–Arg211 in WT vs R211C; Ser346–Arg351 in WT vs A346S). Retention by looser cutoffs holds by construction; retention by stricter cutoffs is the informative part.
