# Supplementary material for: Molecular Dynamics Reveals Structural Consequences of Schizophrenia-Associated SLC6A1 Variants
Source: Int J Mol Sci. 2026 Oct 31;27(21):9793. doi: 10.3390/ijms27219793 (PMC13763465; doi:10.3390/ijms27219793)
Supplement: Supplementary file 1 [file ijms-27-09793-s001.zip › TableS5.pdf]

Table S5. Similarity of per-replica gMI networks within and between systems. Each of the 16 trajectories gave one network on a common set of 10,102 candidate residue pairs. For every pair of replica networks the mean absolute error (MAE) and the Pearson correlation of the unthresholded gMI vectors were computed; the table gives the median of each comparison class.

Comparison	Replica pairs	Median MAE	Median correlation
WT – WT	within system	0.0672	0.8111
R211C – R211C	within system	0.0806	0.7587
A305T – A305T	within system	0.0683	0.8094
A346S – A346S	within system	0.0925	0.7765
WT – R211C	WT versus variant	0.0744	0.7887
WT – A305T	WT versus variant	0.0702	0.7955
WT – A346S	WT versus variant	0.0805	0.7705

Values for WT versus variant (MAE 0.070–0.081; correlation 0.770–0.795) lie within the range found between replicas of a single system (MAE 0.067–0.092; correlation 0.759–0.811). gMI quantifies statistical dependence between residue fluctuations; the table makes no statement about signal transmission or transport.
